# Weeding out the information: an ethnographic approach to exploring how young people make sense of the evidence on cannabis

**DOI:** 10.1186/1477-7517-10-34

**Published:** 2013-11-27

**Authors:** Barbara M Moffat, Emily K Jenkins, Joy L Johnson

**Affiliations:** 1 School of Nursing, University of British Columbia, 302-6190 Agronomy Road, Vancouver, BC V6T 1Z3, Canada

**Keywords:** Cannabis, Youth, Scientific evidence, Public health recommendations, Knowledge translation

## Abstract

**Background:**

Contradictory evidence on cannabis adds to the climate of confusion regarding the health harms related to use. This is particularly true for young people as they encounter and make sense of opposing information on cannabis. Knowledge translation (KT) is in part focused on ensuring that knowledge users have access to and understand best evidence; yet, little attention has focused on the processes youth use to weigh scientific evidence. There is growing interest in how KT efforts can involve knowledge users in shaping the delivery of youth-focused public health messages. To date, the youth voice has been largely absent from the creation of public health messages on cannabis.

**Methods:**

This ethnographic study describes a knowledge translation project that focused on engaging young people in a review of evidence on cannabis that concluded with the creation of public health messages generated by youth participants. We facilitated two groups with a total of 18 youth participants. Data included transcribed segments of weekly sessions, researcher field notes, participant research logs, and transcribed follow-up interviews. Qualitative, thematic analysis was conducted.

**Results:**

Group dynamics were influential in terms of how participants made sense of the evidence. The processes by which participants came to understand the current evidence on cannabis are described, followed by the manner in which they engaged with the literature for the purpose of creating an individual public health message to share with the group. At project end, youth created collaborative public health messages based on their understanding of the evidence illustrating their capacity to “weed out” the information. The content of these messages reflect a youth-informed harm reduction approach to cannabis use.

**Conclusions:**

This study demonstrates the feasibility of involving young people in knowledge translation initiatives that target peers. Youth participants demonstrated that they were capable of reading scientific literature and had the capacity to engage in the creation of evidence-informed public health messages on cannabis that resonate with young people. Rather than simply being the target of KT messages, they embraced the opportunity to engage in dialogue focused on cannabis.

## Background

Approaches to addressing cannabis use are fragmented in Canada, which contributes to a climate of confusion regarding its potential harms. On the one hand, advocates of the substance promote its beneficial properties, while those in opposition focus on the harms associated with the drug. Indeed cannabis is a complex substance with blurred boundaries between its medicinal properties and recreational appeal [1]. Although recreational cannabis use is illegal in Canada, it remains the most widely used illicit drug particularly among young people. Health Canada data indicate that young people are experimenting with cannabis at a younger age than in the past, with the average age of first use currently at 13.7 years [2].

In Canada, there is growing support among law makers and health professionals [3], as well as the general public [4] for changes that will permit the regulation of cannabis in a climate where current approaches to criminalizing the substance are considered counterproductive to overall public health. The support to regulate cannabis in neighboring American States of Washington and Colorado in the 2012 United States election has added fuel to this issue in Canada. Within this environment of polarized views on the substance, little attention has focused on how young people come to understand the potential harms and benefits related to cannabis use.

Navigating the abundant research on cannabis is a continuous exercise in weighing the best evidence. Although the adverse health effects of cannabis use have been studied extensively [5-7], some evidence remains inconclusive which adds to the challenge of addressing the harms related to this drug [8]. Cannabis is a complex substance with differing ratios and effects of its psychoactive components, tetrahydrocannabinol (THC) and cannabidiol (CBD) [9]; to date, however, the psychopharmacology of this substance has been largely under researched. Furthermore, scientific literature supports contradictory evidence. For example, the anxiolytic properties of CBD are well documented [10] and recently have been noted to benefit individuals with schizophrenia [11,12]. In contrast, other findings link cannabis use and psychosis [5,13-15]. Translating similar conflicting evidence to make it accessible is no easy task [16]. In the popular media, cannabis is often depicted in a way which distills and distorts scientific evidence to highlight findings that promote its salutary aspects. As a result, some young people have come to view cannabis as therapeutic [17,18]. An in-depth understanding of how young people make sense of the contrary scientific evidence on cannabis is lacking in an era and social context where they are exposed to diverse sources of information.

An absence of consistent messages and harm reduction strategies related to cannabis has particular implications for young people as they encounter opposing information and make choices regarding its use. Within the Canadian context, young people receive adult-driven public health messages emphasizing the harms of cannabis, yet frequently hear about permissible medicinal use and are exposed to an environment where recreational use among peers and adults is common. Adolescence represents a critical developmental period for targeting substance use prevention and harm reduction strategies given that experimentation with substance use coincides with this stage of development. If our intention is that young people minimize the harms related to cannabis use, an understanding of how they engage in weighing the evidence and making decisions regarding use is indicated.

Approaches to addressing the topic of cannabis are inconsistent in school settings in Canada. A dominant abstinence discourse prevails in some high school communities with well-intended anti-drug messages to refrain from cannabis use altogether. Relying solely on such messages, however, may be ineffective as many young people have started to routinely use cannabis by their early teenage years. Other high school communities convey messages emphasizing that cannabis use will not be tolerated during the school day, which some youth interpret as an attempt to avoid addressing the topic directly [19]. In short, open dialogue and balanced discussion about cannabis with young people is lacking, resulting in minimal engagement to explore their understandings of the risks associated with cannabis.

Reducing the potential harms related to cannabis use requires a planned process of disseminating information and engaging in dialogue regarding the reasons for use and the associated risks based on the best evidence available. The growing field of knowledge translation (KT), which is aimed at promoting the utilization of best evidence to improve health outcomes, provides a useful lens for informing efforts to reduce the risks associated with cannabis use among young people. While KT is a broad field encompassing a range of approaches, there is heightened interest placed on the importance of utilizing collaborative methods, engaging end-users of knowledge throughout the KT process [20,21]. Referred to as interactive or integrated KT, this type of approach is seen to enhance the relevance and meaning of research evidence among knowledge users [22]. Although the decision to use an integrated KT strategy should be based on the purpose of the research and desired outcomes, it may provide a particularly useful approach for enhancing the utilization of evidence among young people given that “children do indeed interpret their worlds differently from adults; they have distinctly different perspectives, values and understandings about all sorts of things…” [23] p. 318. However, while KT provides a mechanism for enhancing youth awareness of best evidence on the harms associated with cannabis use, to date, there has been a reticence to share such evidence with young people within the school context. As a result, there is a lack of understanding regarding young people’s ability to actively engage in the KT process — to critically weigh or appraise scientific evidence, or to be involved in the development of public health messages targeting youth; a gap we seek to address in our research.

It is clear that youth require evidence-based, culturally appropriate strategies that can effectively convey the risks associated with cannabis use. In this KT study, we engaged with young people in a supportive academic environment to foster the development of basic research and critical thinking skills with the goal of enhancing youth capacity to navigate the scientific and lay literature pertaining to cannabis. The purpose of this project was twofold: 1) to understand the processes by which young people work collaboratively to make sense of evidence on cannabis and, 2) to understand how youth engage with the literature for the purpose of contributing to the public health dialogue on cannabis.

## Methods

An ethnographic approach was used in this study to observe how young people worked with cannabis-related materials, synthesized information, and interacted with peers during group activities. An ethnographic approach focuses on complex and multilayered practices and the meanings attached to processes and practices utilized [24]. Ethnographic methods are well suited for providing a holistic approach to explore a phenomenon, in our case, how participants made sense of scientific evidence on cannabis and created public health messages, and presenting this information from participants’ perspectives [25].

Ethical approval was obtained from the Research Ethics Board at the University of British Columbia. Information about participating in this study was circulated widely within high school communities. Our sample consisted of two groups and included a total of 18 young people (10 females and 8 males), aged 15 to 18, who were hired to participate in weekly study sessions. Most participants had never tried cannabis (n = 12), a few had tried on several occasions (n = 3) while others used it regularly (n = 3). The first group took place over a six-week period (n = 10), and the second group met over the course of eight weeks (n = 8). Group 1 was facilitated by two adult researchers, whereas Group 2 was facilitated by one adult researcher and one youth who had been a participant in Group 1. The sessions were held after school at the University of British Columbia in a conference room transformed into an informal learning environment. The group sessions began with an introduction to the basic topics of research, such as confidentiality and informed consent, and participants signed a consent form which outlined the expectations of the research project (e.g., completing homework, attending all sessions). Weekly activities included reviewing select literature and other sources of cannabis-related messaging as well as preparing an individual evidence-informed public health message.

To facilitate the participants’ skills in evidence appraisal, we engaged them in group learning activities that involved a discussion of materials (i.e., research articles, reviews, lay commentaries) selected by the team on topics such as distinguishing evidence from opinion and balanced from biased content. Participants also attended a library orientation that focused on conducting literature searches. In addition, the research team provided ongoing support for accessing and interpreting articles related to their personal projects.

Data were collected from a number of sources. Participants maintained reflective logs and presented their evidence-informed public health message at the end of the project. Sessions were audio-recorded and relevant segments were transcribed. Throughout the project, participant observation occurred and extensive field notes were made; the youth facilitator in Group 2 also maintained field notes. A member check was integrated into the analysis that involved sharing excerpts from group discussion, and emerging findings to ensure that the research team was on track with early analysis and to maximize participants’ understanding of the research process. Individual follow-up interviews were conducted with all participants to further explore their reflections on making sense of the evidence on cannabis. Although we draw on different sources of data in this paper, we focus on the observational component of this ethnographic study, thereby supporting the research teams’ interpretations of how participants worked collaboratively to make sense of the evidence and how they engaged with the scientific literature.

Analysis involved a team of three researchers who focused on reviewing and interpreting sections of the data that focused on how youth made sense of the evidence on cannabis. The software program, NVIVO, was used for the purpose of storing and organizing the data. One member of the research team (BMM) was responsible for managing and coding relevant data. Emergent themes and relevant excerpts from the data were discussed with the research team to ensure accuracy of interpretations.

## Results

Reviewing the evidence on cannabis was an uncharted and challenging task for the participants. From the outset, they were enthusiastic vis-à-vis the prospect of learning more about cannabis and the opportunity to participate in ongoing discussion focused specifically on the substance. We begin by turning our gaze to the overarching group dynamics that unfolded within the confines of this research project. Next, we describe the processes by which participants made sense of the current evidence on cannabis, and finally we depict the manner in which they engaged with the literature for the purpose of creating a public health message to share with the group. At project end, youth in Group 2 created collaborative public health recommendations based on their understanding of the evidence. One participant from Group 2 assisted with the development of a brochure showcasing these messages.

### Group dynamics at play

Participants came to the project with different perspectives and experiences. This clearly influenced the dynamics within the group and shaped the process of collaboratively engaging with the evidence. Some of the young people revealed that they knew very little about the substance, while others possessed strong opinions and claimed knowledge based on their personal cannabis use. This made for animated discussions, inspired curiosity for learning, and accommodated diverse perspectives and dialogue focused on the evidence.

Comfort levels and participation during discussion were distinct between both groups. Personal experience with marijuana was a powerful force that was particularly palpable in Group 1. Those who were regular cannabis users were vocal, revealing their use to others early in the process; they also spoke with authority. Those without personal cannabis experience listened intently to the confident and persuasive voice of “knowledge” based largely on personal experience. Subtle silencing transpired suggesting that some participants were unprepared to challenge this self-assured voice. In contrast, the participants in Group 2 were immediately relaxed with one another. This group included several youth who had used cannabis occasionally, but no regular cannabis users.

In both groups, certain participants (including those without personal cannabis experience) spoke with considerable ease. During discussion, these youth made frequent contributions about their understandings of the evidence and were influential players as demonstrated when select content was later picked up and repeated by others. One participant, who had smoked marijuana once, was described by his peers as having “a way of capturing the audience” when sharing his understanding of the evidence. On occasion, articulate and effusive youth shared misinformation unknowingly whereby it was necessary for the research team to provide clarification. Other participants’ contributions regarding the evidence were measured, suggesting a preference for reflection while quietly making sense of the evidence. These youth surprised the team with their sophisticated understanding of cannabis at project end. Despite the serious focus of the sessions, the dynamics also included laughter and playful elements that were most often initiated by the male participants. Humor appeared to be a way to puncture potential awkwardness within this context of different perspectives and experiences.

### Expecting simple answers

Many participants entered into an examination of the scientific literature with the notion that there would be clear, straight forward answers in the research on cannabis. This was based on the assumption that this body of science was firmly established. As a result, many looked for single causes in research findings and pursued consistent and concrete results. Within little time, however, they encountered unanticipated and contradictory evidence. Many expressed their frustration with these perceived shortcomings in the evidence that was particularly apparent when researchers acknowledged that findings remained “inconclusive.” Proposed “theories” of benefit and harm undermined any sense of certainty and, for some youth, they were difficult to grasp. In response, many participants became guarded when making sense of the evidence; some reacted with skepticism. For example, with regard to the association between cannabis and schizophrenia, one participant remarked, “it could be this [or] it could be this… and it might not be marijuana that’s causing this, [pause] or it could be.” Another young person was particularly dissatisfied when researchers concluded with the phrase “we don’t know” which was interpreted as “an easy out”.

It’s a classic answer for a complicated question in science …it’s better than saying an answer that could be false or that you don’t really have sufficient evidence to back up. So it’s not necessarily like not a bad answer, well, I want more. (Female, 17, occ. MJ use).

It was also somewhat unsettling for some participants to realize that the evidence was neither as solid nor straight forward as had been suggested when exposed to abstinence styled public health messaging. At the same time, it was as if a light switch had been turned on; most were beginning to appreciate the complexity surrounding the substance and were intent on unraveling the puzzle so as to gain more certainty. After reading one academic article, one young woman noted in her log, “the claim that I found interesting was the negative effect on memory may depend solely on the strain [of cannabis].” These youth embarked on the challenge of unpacking the evidence on cannabis to gain more clarity.

### Wrestling with uncertainty

Participants engaged with and “puzzled” over contradictory and inconclusive findings. In addition, unexpected evidence challenged what young people had previously held to be true, which was an obvious source of confusion. Certain findings were simply labeled “bizarre,” pointing to the level of surprise for some participants. For one young man, firm beliefs about the health harms of cannabis were exposed and needed to be re-examined when confronted with evidence suggesting that smoking tobacco was more harmful than cannabis. A female participant commented on the evidence related to cannabis use and driving noting how it was “kind of hard to wrap your head around” some of the evidence adding, “Marijuana impairs your psychomotor performance, so why would some drivers actually improve their performance?” Findings could not be taken at face value. Participants’ efforts and abilities to absorb new information required inquisitiveness and diligence.

Use of conditional language added to existing uncertainty. Specifically, the terms “could” and “may” that frequently appeared in scientific materials were deemed unsatisfactory and vague. In addition, the concept of “risk” was elusive and hard to comprehend. Weighing balanced evidence that explored risk was particularly challenging for those who had previously aligned themselves with strong beliefs that cannabis was either safe or harmful. As such, one young person, a fervent advocate for the use of vaporizers, initially had difficulty accepting that there were risks associated with “vaporizing” cannabis. In contrast, those who had always seen it as a harmful substance struggled to acknowledge the value of purported medicinal benefits.

Some participants came to understand that there were degrees of evidence when sorting through “information.” One young person noted the importance of being able to distinguish between “facts, kind of facts, and somewhat factual.” Communicating their confusion, emerging understandings and insights of the evidence with one another appeared to be a helpful outlet as participants recognized a shared uneasiness with the uncertainty in the literature.

### Relaxing with ambiguity

Over time, we observed that most participants developed the ability to consider opposing findings when making sense of the evidence by applying varying degrees of critical thinking. Most were gaining skills in recognizing bias and valued balanced reporting in the literature that they were encountering.

Participants settled into a position where they proposed that cannabis “affects everybody differently”, making it a substance that was “not black and white”. This indisputable stance appeared to bring some relief. Having reviewed select evidence, they had gained a level of confidence and reflected on how they understood the status of the evidence. The limitations were underscored with claims that “everyone said there needs to be more research”. One youth concluded that “confounding factors can contribute to the results”. They readily acknowledged that the topic of cannabis was “so controversial”. Having explored the literature and weighed the evidence, they felt justified with their position in this middle zone where cannabis was perceived as neither good nor bad.

I thought doing the research would kind of help us find the ‘yes’ and the ‘no’s, but it actually didn’t, it made us more confused. But we did learn more of the why it could be ‘yes’ or it could be a ‘no’. So, I think those are really valuable towards finding the conclusions. (Female, 16, non-user).

Despite this lack of certainty in the midst of inconclusive and conflicting evidence, the exercise of inquiry was deemed to be worthwhile.

Most youth who came into the project thinking that cannabis was “all bad”, no longer believed that was the case at project end, an unanticipated finding for the research team. Furthermore, those who initially held extreme beliefs about cannabis as either a good or bad substance appeared at ease with their uncertainty. Participants had gained an appreciation for the complexity surrounding the evidence on cannabis. Most had developed basic critical thinking skills and were better able to identify bias and unreliable sources of information. As a result, participants were able to relax within the scope of uncertainty that was present in the literature.

### Approaches to reading the literature

Reading the scientific literature involved encountering new terminology and interpreting a “different” style of writing initially described as “hard to read/understand due to the vocabulary and complex sentence structures”. Participants soon developed strategies for “weeding out the information”. The degree to which participants remained engaged in the activity of careful reading varied over the course of the project. Two distinct styles became apparent within several weeks: effortful engagement and intermittent engagement. Although some participants drew on both approaches depending on the material at hand, most relied primarily on one style. Of note, there was no association between personal cannabis use and style of engaging with the literature.

#### Effortful engagement

With the first approach, participants became immersed in a methodical sorting through the evidence on cannabis because they were curious about the topic and wanted to learn. They demonstrated perseverance when grappling with challenging and unfamiliar materials, highlighting articles, making notes, and developing systems for organizing the new information. One youth routinely read articles twice and was purposeful in her approach.

The first time was sort of to get a feel of it and the second time was to pick out key ideas, to pick out words that I didn’t know, to pick out any questions I might have about the article … to focus more on bias. (Female, 17, non-user).

This approach was characterized by enthusiasm for focused learning about the evidence on cannabis, and curiosity about the topic selected for their final research project.

At times, this type of engagement was ephemeral. For one young man, it occurred in spurts based entirely on his interest in specific materials. One research article addressed the debate on measurement issues and cannabis which had sparked his curiosity. After reading it thoroughly, he was articulate in sharing his understanding of the different points of view represented in the article and accompanying commentaries. However, his level of engagement was not sustained when it came to his final presentation, reflective of the second style, intermittent engagement, highlighting the ebb and flow of effort observed with some participants when tasked with engaging with the literature.

#### Intermittent engagement

A second style involved a ‘cherry picking’ approach to reading materials on cannabis. Some young people claimed to already have the answer, whereas others appeared generally capable but uninterested in investing the necessary time. This expedient approach was characterized by “skimming” the literature, all the while expressing confidence in “knowing what to look for” in order to “skip through to the important parts”. One participant elaborated on selecting what to read.

I pick out the parts that I find interesting. And I read those and then I also look through the graphs first because they’re well organized and interesting and I find the relevant sections of text that actually elaborate on those graphs…. I can form my own thought process because I’m reading through it in my own way. (Male, 17, occ. MJ use).

Based on a superficial and partial read, conclusions were drawn quickly. Some entered into the literature, determined to find evidence to support what they already believed and not pursue that which challenged it. On occasion, errors were made as a result of relying on this approach.

### Group outcomes

Despite the challenges that all participants experienced when making sense of the scientific evidence on cannabis, collaborative public health messages were created by participants in Group 2, reflecting language that was concrete and direct. There was a palpable sense of accomplishment that the group had made sense of some evidence on cannabis.

1. It is better to stay abstinent than to suffer the potential consequences.

2. It’s best not to resort to marijuana when life isn’t going well. There is always help available.

3. Initiating cannabis use before adulthood is a lot more dangerous than beginning at a later age.

4. Marijuana affects everyone differently, both physically and mentally. Know what you’re gambling with when using marijuana.

5. If you do choose to use it, make sure it only impacts your life and not the lives of others.

6. Know your source. There may be more in the dose than just marijuana.

7. The higher the dosage, the more severe the impairment.

8. Know the risks, make informed decisions, use responsibly.

As one young person acknowledged, “Public health messaging is focused more and more around knowledge and making your own decision from this knowledge, and less around scare tactics”. Accordingly, these participants had created balanced public health messages based on their shared knowledge on the topic of cannabis following a review of the evidence. It is worth noting that the above public health messages were produced by a group of young people with a low rate of cannabis use. No doubt comparable messages by youth counterparts who use cannabis regularly would encompass a more permissive tone.

How participation in this study would inform decision-making about whether or not to use cannabis in the future was not the goal of this study. That said, at the end of the project, most participants who had not used cannabis conveyed a resolve to “avoid marijuana at all costs”. Participating in this project also influenced self-reflection for several participants who did use cannabis regularly. As one young man noted, “I now feel more cautious in my approach to pot”.

In keeping with a KT approach, the collaborative, youth-driven public health messages were assembled into an information brochure, a process which involved substantial input from a Group 2 participant; these resources were later made available to youth prevention workers based in Vancouver high schools. Responses to these brochures have been positive and, consistent with other KT research projects, demonstrate the enhanced applicability of findings that result from involving the end-users of evidence throughout the entire KT process [26]. One youth worker shared, “I really like the messaging” adding “the layout would work really well for the type of work we do. It gives 8 solid points - great youth voice quotes, and reminds us what is most important to youth.... how the message is communicated”. Another youth worker identified how the brochure supports initiating critical dialogue on cannabis use, which to date, has been largely absent in schools , adding “it helps that it's through youth voice and not the usual adult or health authority”. Requests for additional brochures from several local high schools have resulted in needing to print additional copies. It also points to the dearth of culturally relevant materials available on cannabis to support balanced dialogue on the topic within school settings. Although no formal evaluation of brochure has yet occurred, it is clear that the brochure has been a welcome resource.

By project end, participants conveyed continuing enthusiasm to learn more about cannabis, demonstrating that their interest in the evidence was alive and well. One young man suggested, “Besides making our own messages for teens, I feel that we can use our research to try and reach out to programs such as D.A.R.E. to help them improve their courses”. They expressed appreciation for the opportunity to have engaged in research focused on cannabis that had “broadened knowledge” and “opened their eyes” to the challenges in drawing “definite conclusions”. Attitudinal shifts towards the substance were expressed from a position of “strictly opposed and ignorant” to “more educated” and able to “consider both sides”. And one young man who used cannabis on a regular basis noted, from here on, he would be “more careful checking the credibility of facts”. Most were visibly eager to deliver their personal health message and to participate in discussion about a topic that was perceived as “controversial, misunderstood, demonized” and rarely talked about in a non-judgmental forum. One young woman proposed, “Just keep the conversation open… teens love to express themselves given the chance”.

## Conclusions

There are clear benefits to understanding how young people make sense of the literature on cannabis. Most youth in this project were capable of reading scientific literature, making sense of content and weighing potential risks related to cannabis use. Given the status of the current evidence on cannabis as well as the abundance and accessibility of information, can we expect youth to reach an unwavering understanding of the associated risks? While it is not possible to monitor how youth access information on cannabis, it is possible to support them in the review of credible sources, and in so doing, foster capacity for the critical examination of evidence. Importantly, our study findings point to untapped opportunities to challenge beliefs on cannabis and encourage reflection upon possible misunderstandings.

The group setting provided an important context that facilitated some participants’ understandings of the evidence of cannabis, albeit with prominent group dynamics at play. There was clearly much value from the discussion component of the project, a time to reflect on and share understandings of the evidence within the group setting. Creating and supporting this environment where youth were able to participate in facilitated cannabis-related discussion with peers and not be judged for held views or personal cannabis practices was well received. Participants were eager to discuss opposing research findings, highlighting the pros and cons of cannabis use, an opportunity that did not exist within the school setting. Building similar opportunities for in-depth discussion on cannabis to encourage young people to think critically about the evidence would foster meaningful dialogue.

The different approaches applied to making sense of the evidence are hardly a surprise given the range in personal learning styles and abilities to take in new information. In our study, we intentionally focused on youth aged 15 and older, recognizing that younger people (i.e. those in Grades 8 and 9) need structure and would likely have encountered additional challenges with the demands of the project. Participants were motivated to make sense of the evidence when they were passionate about a specific topic. However, Gasser [27] notes the double edge sword of knowledge and expertise readily available on the internet, specifically “how the absence of traditional gatekeepers engenders a complicated information landscape, capable of facilitating honest exchange and empowerment as well as danger and harm” (p. 40), hence the urgency of taking into consideration *what* information young people are now able to uncover. Indeed, this highlights the need to encourage access to and support the uptake of credible information on cannabis.

Admittedly language use remains a challenge in public health [28]; conveying precise and accurate “risk” information to youth and adult populations regarding cannabis is no easy task when much remains unclear [29]. Our study findings point to the importance of using clear and precise terminology. Ambiguity can be off putting for young people with the use of terms such as “could” and “may”, perceived to be indicative of a failure to take a stand by some youth in our study. Not surprisingly, participants carefully selected simple and direct language for their collaborative public health recommendations. How the evidence on cannabis translates into public health messages, including potential risk deserves ongoing attention. Given that proposed “theories” regarding risk were perceived to undermine certainty, they may be beyond the comprehension of some youth. Nonetheless, engaging in meaningful discussion about and reflecting upon possible risks and theories appears to contribute to more sophisticated understandings as was the case for some participants in this study.

With regard to researchers engaged in KT activities, our findings reveal that young people are indeed capable and appreciative of the opportunity to engage in KT activities. Furthermore, given that involving knowledge users in the KT process is seen as an important step towards enhancing the outcomes of this work, youth must be acknowledged and engaged as a central stakeholder group, particularly when it concerns evidence for use by youth populations; young people respond differently to information than do adults [23].

Finally, project findings revealed how young people can contribute to the public health dialogue on cannabis. Creating accurate and credible public health recommendations is undeniably a challenge, yet the youth in our study were able to do so. Currently in Canada, there is little consensus on appropriate public health messages for this illicit yet widely used substance. Recommendations or “Lower risk guidelines” for cannabis use were created for adult populations [30] and implicitly for youth and young adult populations in a “Taking Care with Cannabis”, brochure [31]; both were written by adults without input from young people. Including youth voices and perspectives on culturally relevant public health recommendations has the potential for reaching the youth population in a credible fashion. For most youth who are recreational cannabis users, cannabis is harmless, a rite of passage that ends as young people settle into adults lives and careers [6]. Balanced public health dialogue regarding potential risks of cannabis based on the best evidence must be part of health education for it to be deemed believable.

This study is not without limitations. Most participants were clearly motivated as demonstrated by their willingness to participate in an after school activity that required additional reading and project work; most were high achievers academically. Given this small sample, our findings are not representative of all youth. In many ways, participants reflected the demographics of Vancouver; for some, English was not their first language which may have added a layer of difficulty with comprehension of some materials. One participant from Group 1, who encountered challenges finalizing his research project, did not attend the final session and was lost to follow-up. Gordon [32] notes that in ethnography, data is generated by researchers rather than collected. Obviously, the team could only make observations when youth were physically present. Although some research logs contained rich data that shed light on making meaning of the evidence on cannabis, it was not possible to observe what transpired when youth were engaged in making sense at home.

As adults, it is a challenge to understand the perspectives of young people. The aim of this study was to attempt to understand how young people make sense of the evidence on cannabis. Our study findings reveal the abilities of some young people to critically review the evidence and to contribute to public health and harm reduction messaging. As Reist proposes, health literacy in the domain of drug education is a resource or asset, and a precursor for healthy action [33]. Adults involved in the field of public health must not underestimate the capabilities of young people.

## Abbreviations

DARE: Drug abuse resistance education; KT: Knowledge translation; B.C.: British Columbia.

## Competing interests

The authors declare that they have no competing interests.

## Authors’ contributions

BMM led the conceptualization, design, project coordination, analysis and writing of this manuscript. EJ participated in coordination of the study, contributed to analysis and interpretation of the data and writing of the manuscript. JJ is the principal study investigator, contributed to the conceptualization, design, analysis and interpretation of the data, and writing of the manuscript. All authors read and approved the final manuscript.
